# A nomogram for predicting neonatal apnea: a retrospective analysis based on the MIMIC database

**DOI:** 10.3389/fped.2024.1357972

**Published:** 2024-09-05

**Authors:** Huisi Huang, Yanhong Shi, Yinghui Hong, Lizhen Zhu, Mengyao Li, Yue Zhang

**Affiliations:** Department of Paediatrics, The Affiliated TCM Hospital of Guangzhou Medical University, Guangzhou, Guangdong, China

**Keywords:** logistic regression, nomogram, neonatal apnea, MIMIC database, retrospective analysis

## Abstract

**Introduction:**

The objective of this study is to develop a model based on indicators in the routine examination of neonates to effectively predict neonatal apnea.

**Methods:**

We retrospectively analysed 8024 newborns from the MIMIC IV database, building logistic regression models and decision tree models. The performance of the model is examined by decision curves, calibration curves and ROC curves. Variables were screened by stepwise logistic regression analysis and LASSO regression.

**Results:**

A total of 7 indicators were ultimately included in the model: gestational age, birth weight, ethnicity, gender, monocytes, lymphocytes and acetaminophen. The mean AUC (the area under the ROC curve) of the 5-fold cross-validation of the logistic regression model in the training set and the AUC in the validation set are 0.879 and 0.865, respectively. The mean AUC (the area under the ROC curve) of the 5-fold cross-validation of the decision tree model in the training set and the AUC in the validation set are 0.861 and 0.850, respectively. The calibration and decision curves in the two cohorts also demonstrated satisfactory predictive performance of the model. However, the logistic regression model performs relatively well.

**Discussion:**

Our results proved that blood indicators were valuable and effective predictors of neonatal apnea, which could provide effective predictive information for medical staff.

## 1 Introduction

Neonatal period is a critical stage for promoting the growth and development of children and preventing future diseases. However, it is a complex process for adaptation from the intrauterine to extrauterine environment, with up to 10% of the newborns requiring certain clinical intervention at birth (1).Once the newborn is exposed to the external environment and begins to breathe, oxygen level in the blood is higher than that in the uterus. Oxygen is a potent dilator of the lungs. The decreased resistance of pulmonary vessels increases pulmonary blood flow, subsequently promoting oxygen delivery throughout the body. With oxygenation enhanced, calcium channels in the smooth muscle of the ductus arteriosus are activated, thereby influencing the pump function of the heart (2). Apnea is the most common manifestation of immature cardiorespiratory regulation in the neonates (3) and can lead to neonatal hypoxemia and hypercapnia (4), which increases the risk of brain injury and neurodevelopmental impairment in the infants (4–6). Although there is no significant data demonstrating the association between apnea and sudden infant death syndrome (6), apnea was a precursor to neonatal asphyxia and could lead to irreversible central nervous system damage according to a previous animal research (7). Regardless of the type and level of apnea, recurrence and long duration of apnea is associated with neurological disease and mortality in neonates.

The breathing of high-risk neonates can be continuously monitored in Neonatal intensive care units (NICUs). However, medical staffs are often distracted by large numbers of newborns and alarms from other events. Early identification or prediction of the risk of neonatal apnea can allocate medical resources in a more reasonable way, and early intervention can be provided to the neonates with high risk of apnea, which has significant clinical significance. There were numerous studies on the monitoring of neonatal apnea and bradycardia based on machine learning algorithms (8). However, few studies focused on the early identification and prediction of neonatal apnea. Two research studies established a predictor for neonatal apnea based on sophisticated algorithms of machine learning (9, 10). The “black box” effect cannot be avoided in these machine learning algorithms, and thus it is challenging to interpret the operation process and quantify the risk of each feature.

Therefore, in order to improve the interpretability and simplicity of the model, a logistic regression algorithm was used to establish a nomogram model according to indicators in the blood test and medication information of the neonates to rapidly predict the risk of neonatal apnea. In addition, we build a decision tree model to compare with the nomogram model. ROC curve, decision curve and calibration curve were plotted using the predicted results and the results observed in clinical practice to test the performance of the model. The findings showed that the prediction performance of the model was satisfactory.

## 2 Methods

### 2.1 Data sources

The data in this study were obtained from the MIMIC IV (https://physionet.org/content/mimiciv/1.0/) database for intensive care medicine. MIMIC IV is a comprehensive dataset containing the demographic information, laboratory results on admission and during ICU stay, diagnosis, medication, survival status, scores of each items, etc. for each participant. This present study included all neonates registered in MIMIC IV who were admitted to the ICU. The demographic information, indicators of laboratory test, and medication of the included neonates were collected: (1) Basic patient information: race, sex, insurance status, gestational age (GA), weight; (2) Indicators of laboratory tests: white blood cells (WBC), basophils, eosinophils, lymphocytes, monocytes, neutrophils, band cells, hematocrit value, hemoglobin, mean corpuscular hemoglobin (MCH), mean hemoglobin concentration (MCHC), mean corpuscular volume (MCV), platelets, red blood cells (RBC), red blood cell distribution width (RDW), total bilirubin (TBIL), direct bilirubin (DBIL), indirect bilirubin (IBIL); (3) Medication: acetaminophen, vancomycin, other antibiotics; (4) Blood culture: Staphylococcus aureus (+). The value measured for the first time after birth was used as the indicators of the laboratory tests. The outcome variable was defined as the occurrence of apnea during hospitalization. Neonatal apnea was defined as apnea ≥ 20 s or apnea ≤ 20 s accompanied by bradycardia (<100 bpm), cyanosis, pallor, and/or obvious hypotension (11). Neonatal apnea is defined by the ninth and tenth versions of the International Classification of Diseases (ICD-9 and ICD-10).

### 2.2 Statistical analysis

Before the analysis, the dataset was randomly divided into training dataset and validation dataset at a ratio of 7:3. The training dataset was used for model establishment, and the validation dataset was for model validation. The demographic and clinical features of the patients were assigned into training and validation datasets for description. Categorical variables were expressed as percentiles (%). Continuous variables with normal distribution were expressed as mean and standard deviation [Mean (±SD)], and those with abnormal distribution were expressed as medians and quartiles [Median(IQR)].

Training dataset was used to feature selection, including univariate and multivariate analysis. In univariate analysis, chi-square test was applied to compare the differences between categorical variables in the two groups, and the one-way ANOVA or rank sum test was applied to compare the differences between continuous variables in the two groups. The variables with statistically significant difference in univariate analysis were subjected to stepwise logistic regression for multivariate analysis. The variables with statistically significant difference in multivariate analysis were subjected to the LASSO regression. To simplify the model, LASSO regression was used to screen the features again, where variables with non-zero coefficients were included in the final logistic regression model and decision tree model. Subsequently, to avoid multicollinearity, we calculated the variance inflation factor (VIF) of the variables in the logistic regression model and excluded those with VIF higher than 5. In the training cohort, 5-fold cross-validation was performed on the model. The performance of the model was evaluated by plotting ROC curve, calibration curve and decision curve. All statistical analyses were performed using R 4.1.2 (The R foundation for statistical computing, Vienna, Austria) (https://www.r-project.org/) and SPSS 26.2. *P* < 0.05 was considered as statistically significant.

## 3 Results

### 3.1 Patient data

Data on 17,376 neonates were retrieved from the MIMIC IV database. After excluding cases with missing data, a total of 8,024 neonates were included in the study, of which 3,069 had apnea. There was no significant difference in each variable between the training dataset (*n* = 5,618) and the validation dataset (*n* = 2,406). The proportion of neonates with apnea was about 38.2% in both datasets (Table 1).

**Table 1:** Data description.

Characteristic	Overall *N* = 8,024	Training Set *N* = 5,618	Verification Set *N* = 2,406	*p*-value
Insurance				0.646
Medicare	12 (0.1%)	10 (0.2%)	2 (<0.1%)	
Medicaid	983 (12%)	693 (12%)	290 (12%)	
Other	7,029 (88%)	4,915 (87%)	2,114 (88%)	
Ethnicity				0.547
White	3,721 (46%)	2,620 (47%)	1,101 (46%)	
Asian	1,027 (13%)	715 (13%)	312 (13%)	
Black	890 (11%)	635 (11%)	255 (11%)	
Other	2,386 (30%)	1,648 (29%)	738 (31%)	
Apnea	3,068 (38%)	2,148 (38%)	920 (38%)	0.998
GA				
>36 weeks	3,753 (47%)	2,615 (47%)	1,138 (47%)	
≤28 weeks	552 (6.9%)	378 (6.7%)	174 (7.2%)	
28–32 weeks	1,212 (15%)	863 (15%)	349 (15%)	
32–36 weeks	2,505 (31%)	1,761 (31%)	744 (31%)	
Unknown	2 (<0.1%)	1 (<0.1%)	1 (<0.1%)	
Weight				0.756
<1,500 g	1,089 (14%)	761 (14%)	328 (14%)	
>2,500 g	4,683 (58%)	3,260 (58%)	1,423 (59%)	
1,500–2,500 g	2,248 (28%)	1,594 (28%)	654 (27%)	
Unknown	4 (<0.1%)	3 (<0.1%)	1 (<0.1%)	
Gender				0.565
Female	3,641 (45%)	2,561 (46%)	1,080 (45%)	
Male	4,383 (55%)	3,057 (54%)	1,326 (55%)	
WBC (10^9/L)	14 [10, 19]	13 [10, 19]	14 [10, 19]	0.07
Basophils (10^9/L)	0.0 [0.0, 0.0]	0.0 [0.0, 0.0]	0.0 [0.0, 0.0]	0.478
Eosinophils (10^9/L)	0 [0, 20]	0 [0, 20]	0 [0, 21]	0.249
Lymphocytes (10^9/L)	176 [5, 493]	179 [5, 486]	162 [5, 507]	0.195
Monocytes (10^9/L)	10 [1, 77]	10 [1, 77]	7 [1, 75]	0.839
Neutrophils (10^9/L)	56 [9, 449]	62 [9, 442]	51 [9, 457]	0.886
Basophils% (%)	0.00 [0.00, 0.00]	0.00 [0.00, 0.00]	0.00 [0.00, 0.00]	0.438
Eosinophils% (%)	1.00 [0.00, 3.00]	1.00 [0.00, 3.00]	2.00 [0.00, 3.00]	0.43
Lymphocytes% (%)	38 [24, 56]	38 [24, 56]	38 [24, 57]	0.522
Monocytes% (%)	7.0 [5.0, 10.0]	7.0 [5.0, 10.0]	7.0 [5.0, 10.0]	0.354
Neutrophils% (%)	48 [31, 63]	48 [31, 63]	48 [31, 63]	0.433
Bands (%)	0.00 [0.00, 2.00]	0.00 [0.00, 2.00]	0.00 [0.00, 2.00]	0.512
Hematocrit (%)	49 [45, 53]	49 [45, 53]	49 [46, 53]	0.639
Hemoglobin (10 g/L)	16.70 [15.30, 18.10]	16.70 [15.30, 18.10]	16.70 [15.30, 18.10]	0.923
MCH (pg)	36.10 [34.90, 37.40]	36.10 [34.90, 37.40]	36.10 [35.00, 37.40]	0.838
MCHC (10 g/L)	33.90 [32.90, 34.80]	33.90 [33.00, 34.80]	33.90 [32.90, 34.80]	0.09
MCV (fL)	107 [102, 112]	106 [102, 112]	107 [102, 112]	0.191
Platelet (10^9/L)	262 [216, 311]	263 [216, 311]	261 [217, 311]	0.866
RBC (10^12/L)	4.63 [4.20, 5.04]	4.63 [4.20, 5.04]	4.62 [4.21, 5.05]	0.916
RDW (%)	16.70 [15.90, 17.60]	16.60 [15.90, 17.60]	16.70 [15.90, 17.60]	0.451
TBIL (mg/dl)	6.20 [4.80, 7.80]	6.20 [4.80, 7.80]	6.20 [4.80, 7.70]	0.776
DBIL (mg/dl)	0.30 [0.20, 0.30]	0.30 [0.20, 0.30]	0.30 [0.20, 0.30]	0.836
IBIL (mg/dl)	5.90 [4.50, 7.50]	5.90 [4.50, 7.50]	6.00 [4.50, 7.40]	0.769
Antibiotic				0.985
No	2,270 (28%)	1,589 (28%)	681 (28%)	
Yes	5,754 (72%)	4,029 (72%)	1,725 (72%)	
STAPH AUREUS COAG +				0.309
No	7,816 (97%)	5,479 (98%)	2,337 (97%)	
Yes	208 (2.6%)	139 (2.5%)	69 (2.9%)	
Acetaminophen				0.725
No	5,149 (64%)	3,612 (64%)	1,537 (64%)	
Yes	2,875 (36%)	2,006 (36%)	869 (36%)	
Vancomycin				0.391
No	7,613 (95%)	5,338 (95%)	2,275 (95%)	
Yes	411 (5.1%)	280 (5.0%)	131 (5.4%)	

### 3.2 Feature selection and model establishment

**Figure 1:**
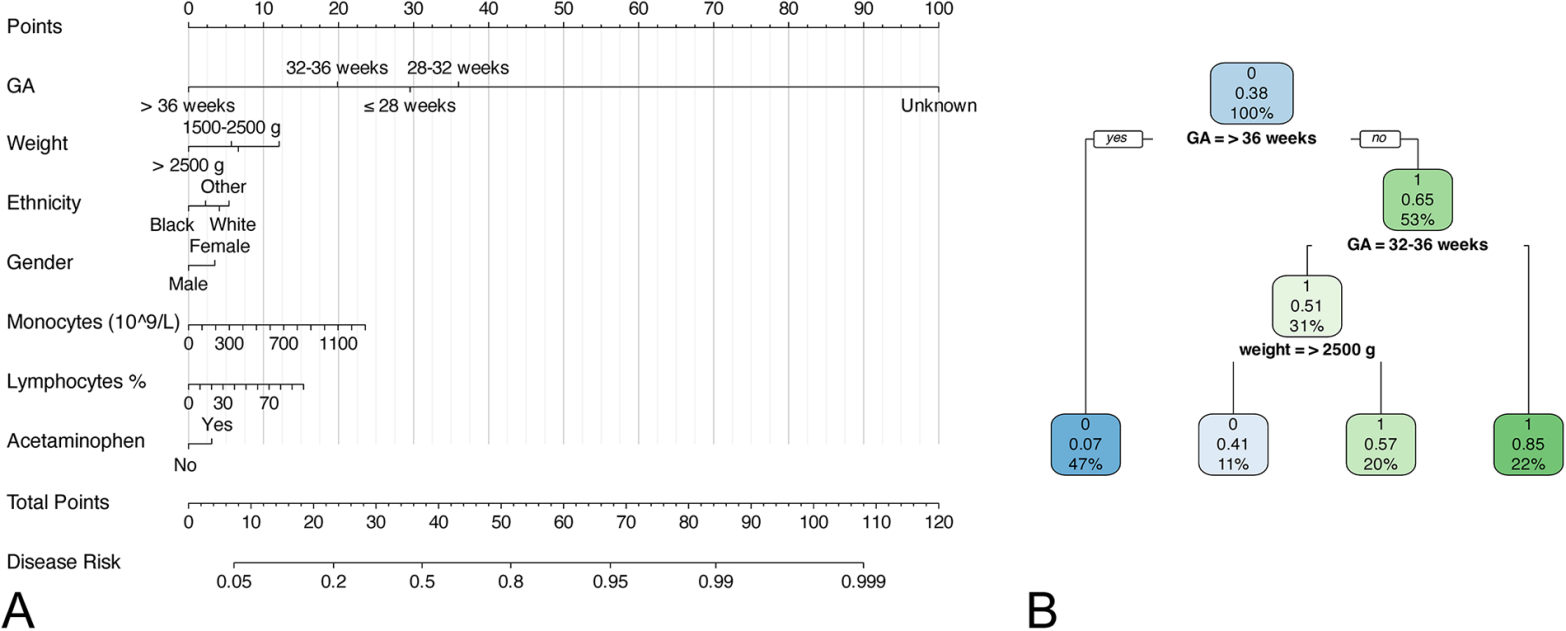
Nomogram and decision tree model for predicting neonatal apnea. **(A)** nomogram; **(B)** decision tree model.

**Figure 2:**
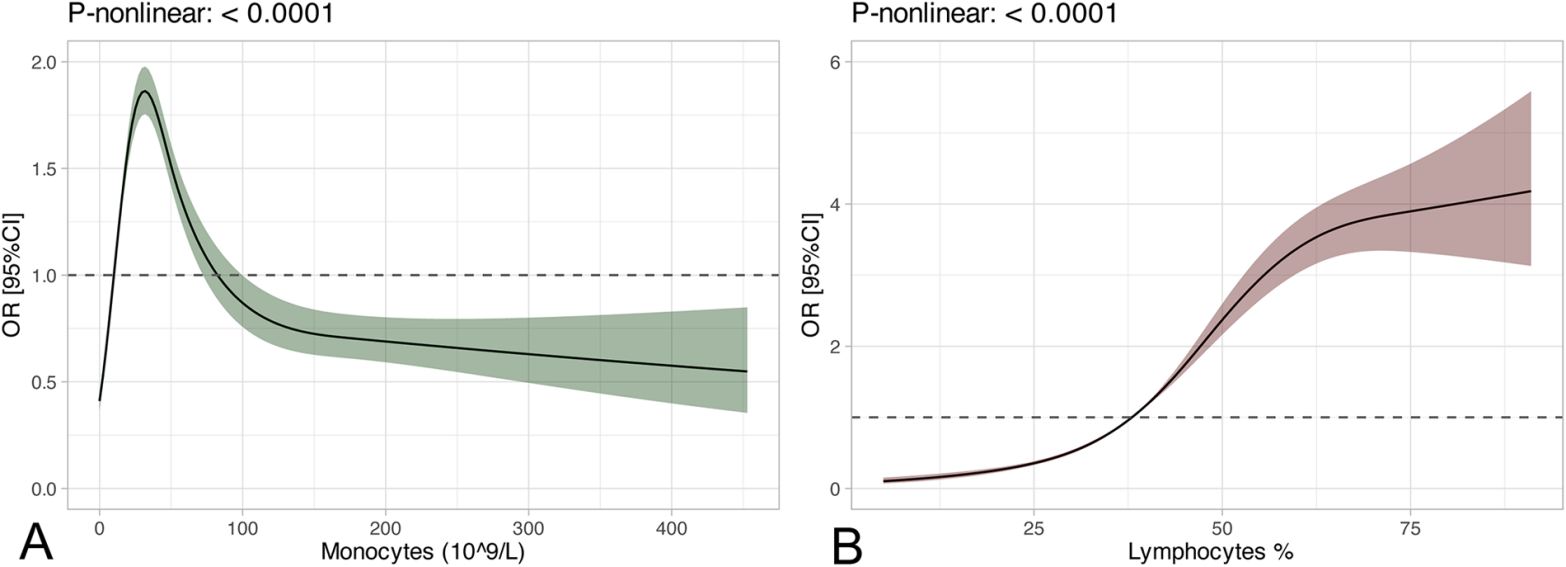
RCS curves. **(A)** RCS curve of the Monocytes; **(B)** RCS curve of the Lymphocytes.

To ensure the simplicity of the model, we included some routine laboratory indicators that are readily available in the ICU. Univariate analysis showed that there was significant difference in 27 of the 32 variables between the neonates with and without apnea. A forward stepwise logistic regression analysis was performed on these significantly different variables. The results showed that gestational age (GA), birth weight, white blood cell count, monocyte count, percentage of eosinophils, percentage of neutrophils, band cell count, mean corpuscular volume, total bilirubin, direct bilirubin, race, sex, antibiotics, acetaminophen, and vancomycin were potential independent risk factors for neonatal apnea (Supplementary Table S1). LASSO regression was used for further screening to simplify the model, and variables with non-zero coefficients in LASSO regression were included in the final logistic regression model and decision tree model (Figure 1), The lambda in the LASSO regression takes the value of lambda with the smallest mean cross-validated error (0.001728152) (Supplementary Figure S1). Seven predictors were finally included: GA, birth weight, ethnicity, gender, monocytes, lymphocytes and acetaminophen (Supplementary Table S2, Figure S1). The variance inflation factors of the variables in the model were all less than 5, indicating a low level of collinearity (Supplementary Table S3). For numeric variables in the model, we performed restricted cubic spline (RCS) analyses to explore potential nonlinear relationships between numeric features and outcomes. The results showed that monocytes (*P* < 0.0001), and lymphocytes (*P* < 0.0001) had significant nonlinear associations with the risk of apnea (Figure 2).

### 3.3 Validation of the model

**Figure 3:**
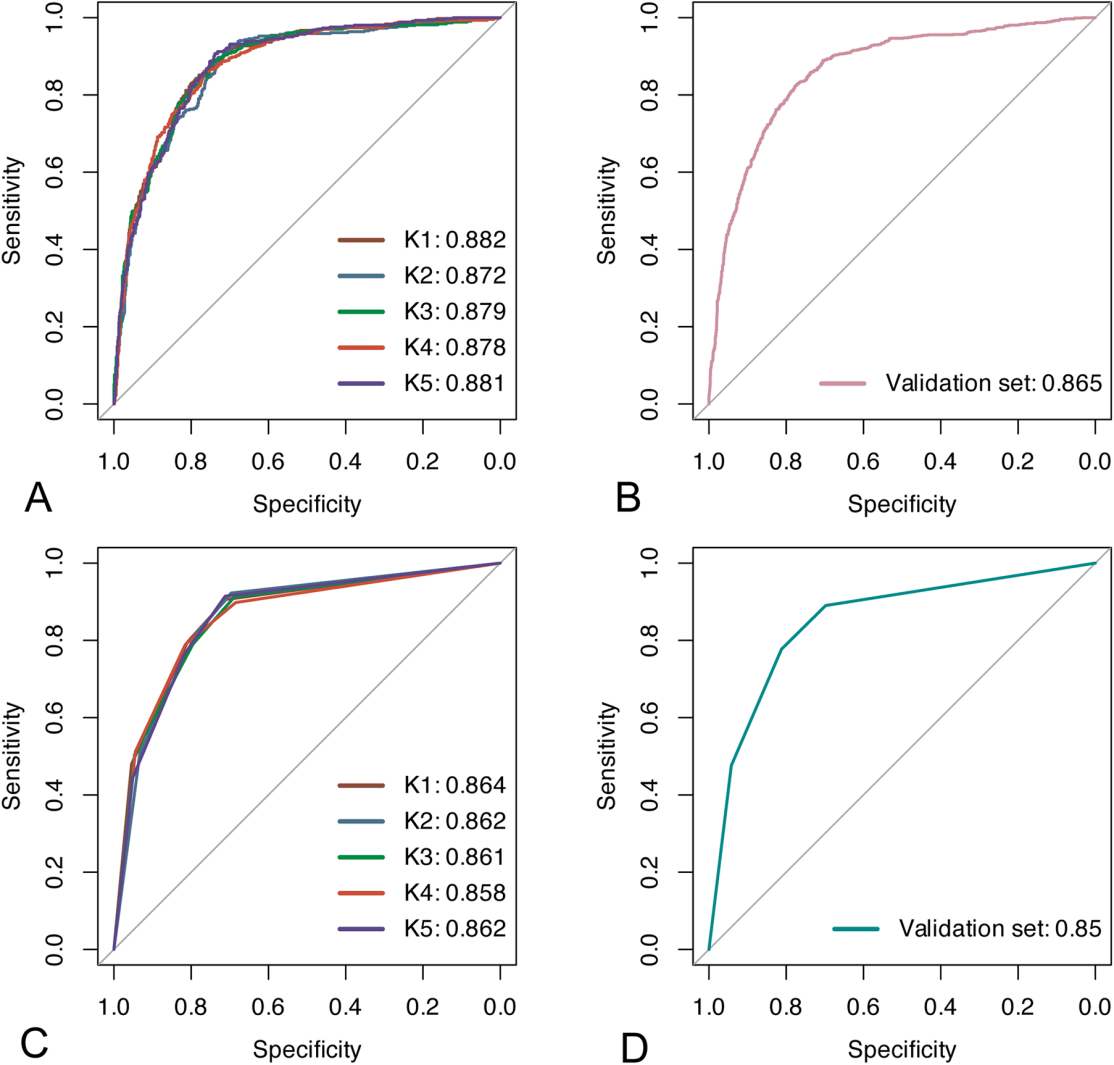
Results of ROC analyses. **(A,B)** ROC curves for the logistic regression model in the training and validation sets; **(C,D)** ROC curves for the decision tree model in the training and validation sets.

**Figure 4:**
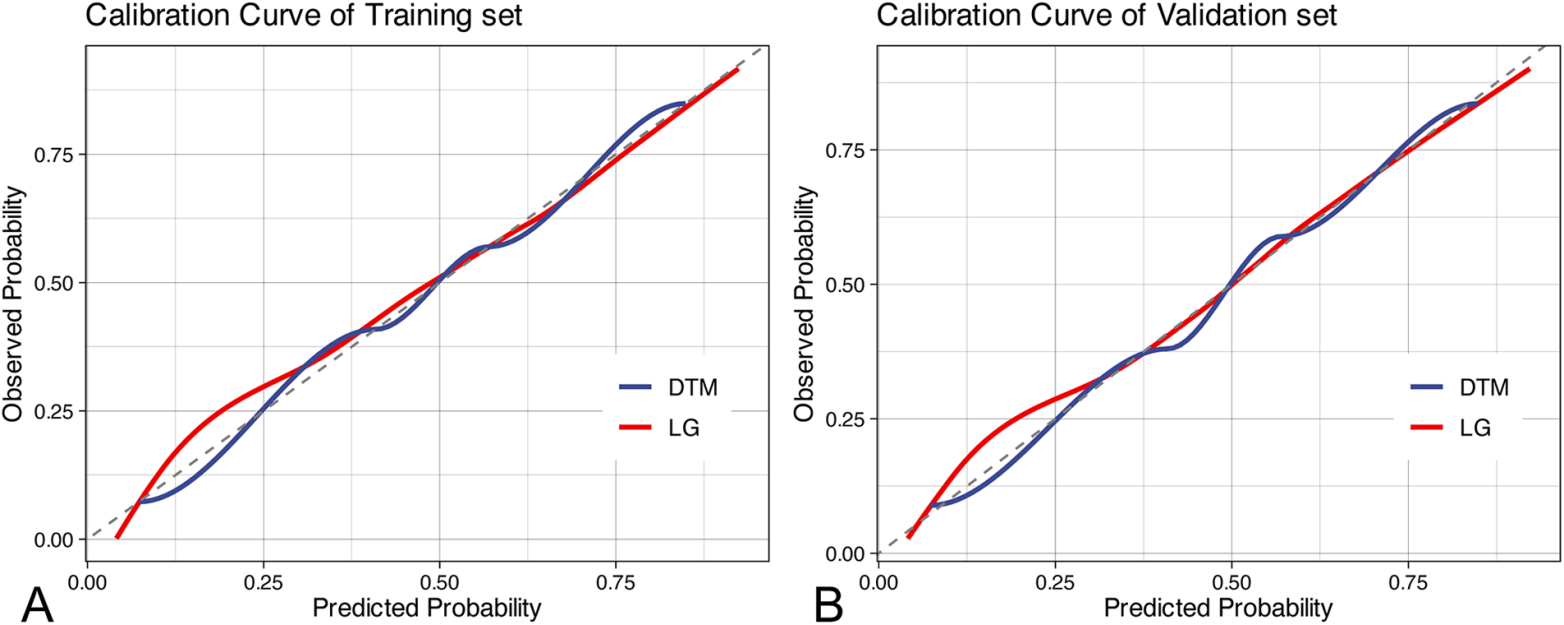
Calibration curves. **(A)** calibration curves in the training set; **(B)** calibration curves in the validation set. DTM, decision tree model, LG, logistic regression.

**Figure 5:**
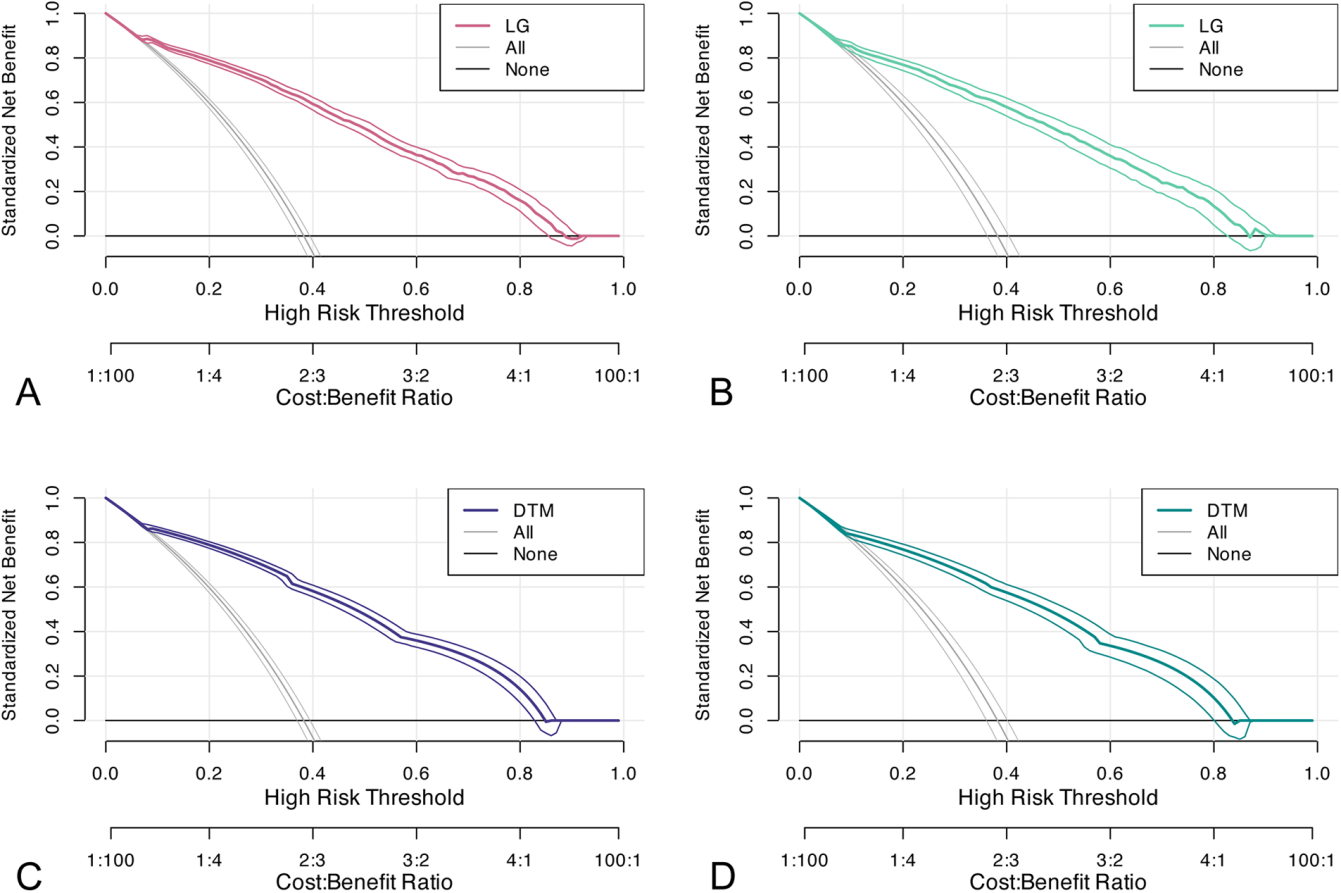
Decision curves. **(A,B)** decision curve of the logistic regression model in the training and validation sets; **(C,D)** decision curve of the decision tree model in the training and validation sets. DTM, decision tree model, LG, logistic regression.

In the training cohort, we performed 5-fold cross-validation on two models. The mean AUC of the 5-fold cross-validation of the logistic regression model in the training set and the AUC in the validation set were 0.879 and 0.865, respectively (Figures 3A,B). The mean AUC of the 5-fold cross-validation of the decision tree model in the training set and the AUC in the validation set were 0.861 and 0.850, respectively (Figures 3C,D). Within the ROC curve, the largest Youden index was used as our optimal cutoff value. In both training cohort and validation cohort, the predicted and observed values were consistent as shown in the calibration curve (Figure 4). Linear regression was fitted by dividing the predicted and observed values of the logistic regression model and the decision tree model into 50 sets. The result of the logistic regression model showed that the intercept in the training set was 0.001 and the slope was 0.997. The intercept in the validation set was 0.017 and the slope was 0.968. The result of the decision tree model showed that the intercept in the training set was -1.055e-16 and the slope was 1.000. The intercept in the validation set was 0.009 and the slope was 0.976. The intercept in both the training and validation sets was close to 0 and the slope was close to 1, indicating that the model is well calibrated. And considerable net benefit was demonstrated in the decision curve (Figure 5). Combined with the results of the ROC, the logistic regression model had a net benefit of 0.640 in the training set when the optimal Youden index was 0.623, sensitivity was 0.886, and specificity was 0.737, for a total of 2,815 children who were at high risk of apnea. The net benefit of the model in the validation set was 0.640, sensitivity was 0.828, and specificity was 0.772, for a total of 1,179 children who were at high risk for apnea. The decision tree model had a net benefit of 0.756 in the training set when the optimal Youden index was 0.609, sensitivity was 0.911, and specificity was 0.698, for a total of 3,003 children who were at high risk of apnea. The net benefit of the model in the validation set was 0.736, sensitivity was 0.777, and specificity was 0.812, for a total of 1,268 children who were at high risk for apnea.

## 4 Discussion

Certain predictors associated with neonatal apnea were identified in this study. The results showed that gestational age, birth weight, ethnicity, gender, monocytes, lymphocytes and acetaminophen were potential predictors for neonatal apnea. A nomogram model and a decision tree model were established based on these factors to predict the occurrence of neonatal apnea. These indicators included in the model are readily available in clinical practice and can be obtained early as soon as new-borns are born, making the clinical prediction of neonatal apnea simple. Calibration curve, ROC analysis and DCA all presented that the nomogram had satisfactory predictive performance. Overall, the logistic regression model performed relatively well.

A few studies have discussed the predictors for neonatal apnea. Among them, Nicola et al. investigated the risk factors of neonatal apnea after immunization in NICU. The study was based on a cohort of 497 neonates, including birth weight, maternal age, feeding status, use of caffeine drugs, and other related indicators in perinatal period. However, the predictive performance of these measures for apnea was not quantified and only indicators related with perinatal period were included without exploring the potential predictive value of other indicators in the study (12). Additionally, other two research teams established predictive models for neonatal apnea. Rudresh et al. established a deep neural network model to predict neonatal apnea based on a cohort of 367 neonates (9). The model included the demographics, indicators related with perinatal period, and physiological parameters. The authors also compared the model with algorithms such as Support Vector Machines (SVM), K-Nearest neighbor, decision tree, and random forest, and the results showed that the deep neural network algorithm was better. By using algorithms such as Gaussian model, another research team established a predictive model for predicting the severity of apnea in preterm infants based on the cardiopulmonary signals and motion features of 10 premature infants. The average AUC of this model was 0.77 and it could predict 83% and 75% of the most severe onset in the training set of 7 infants and the testing set of 3 infants, respectively (13). However, the risk of each feature was not quantified by the algorithms used in these studies, and the results were not visually expressed. Moreover, none of these studies investigated the effect of blood parameters on neonatal apnea. Hence, in order to improve these two shortcomings, we included indicators such as neonatal demographic information, blood parameters, and medication. Logistic regression was used to quantify the risk of each feature, and the corresponding nomogram was plotted. As a common research method in medical research, nomogram can directly demonstrate the risk score of each feature (14–17). The blood indicators and medication were included in our model, and the mean AUC of the 5-fold cross-validation of the model in the training set and the AUC in the validation set were 0.879 and 0.865, respectively, suggesting that blood indicators and medication could also predict neonatal apnea. Our research presented that increased mononuclear cells in blood was associated with an elevated risk of neonatal apnea. Monocyte, an important kind of circulating leukocytes in both innate and adaptive immunity, mainly plays a role in immune defense, inflammation and tissue remodeling (18). Hence, our model may also have a certain warning effect on these events.

Although gestational age and birth weight are physiologically strongly correlated and would normally be expected to exhibit high levels of multicollinearity, in this study we observed variance inflation factors (VIFs) below 5 for both variables, suggesting that there were low levels of multicollinearity in our model. This result may be influenced by several factors. First, our dataset contains a large amount of individual variability, which may have weakened the correlation between gestational age and birth weight. For example, factors such as maternal health status and placental function may have mitigated the multicollinearity between these two variables to some extent (19). Second, we included other covariates in the model, which may have statistically adjusted the association between gestational age and birth weight, thereby reducing multicollinearity (20). In addition, it has been shown that the association between gestational age and birth weight may not always be highly correlated in a given study population, especially in the presence of multiple confounders, which may result in lower than expected VIF values (21). Therefore, we believe that the low level of covariance between gestational age and birth weight in this study is reasonably explainable and does not affect the robustness and explanatory power of the model. However, future studies are still needed to further explore the relationship between these variables in different populations and larger samples to validate our findings.

Although we provided well-performing models for predicting apnea, there are still some limitations of this study that need to be mentioned. First, the study cohort used to establish this model was from the same dataset. Adequate external validation of this model is needed. Second, this study was established based on retrospective data and requires prospective validation in a larger sample. Also, since the database used is based on the US population, the model may not be applicable in regions such as Asia or Africa. Additionally, only two models have been developed in our study, and the applicability of other novel machine learning models in predicting apnea needs to be explored. Eventually, due to the limitations of the public database, we cannot get the specific time of the onset of apnea events, laboratory examinations, and drug administration. Therefore, we will conduct a prospective study to validate the results in the future. However, since these infants live in NICUs, and some laboratory examinations are usually performed on their plantar blood upon their birth, it can be inferred that apnea occurs after the laboratory indicators are obtained. As a result, we think the research is feasible.

## 5 Conclusion

This study proposed a model for predicting neonatal apnea using blood indicators and medication. Our results proved that blood indicators were valuable and effective predictors of neonatal apnea, which could provide effective predictive information for medical staff.

## Data Availability

Publicly available datasets were analyzed in this study. This data can be found here: [MIMIC IV] repository, [(https://physionet.org/content/mimiciv/1.0/)].
