# Supplementary material for: A nomogram for predicting neonatal apnea: a retrospective analysis based on the MIMIC database
Source: Front Pediatr. 2024 Sep 5;12:1357972. doi: 10.3389/fped.2024.1357972 (PMC11410630; doi:10.3389/fped.2024.1357972)
Supplement: Supplementary file 1 [file Datasheet1.pdf]

Supplementary Table S1. Feature selection.

		Univariate Analysis		Stepwise Logistic Regression		
Factor		$\|Z\| / \chi^2$	P	**OR**[1]	**95% CI**[1]	**p-value**
GA	≤ 28 weeks	2334.2	<0.001	—	—	
	28-32 weeks			1.76	1.19, 2.62	0.005
	> 36 weeks			0.06	0.03, 0.09	<0.001
	32-36 weeks			0.38	0.25, 0.59	<0.001
weight	< 1500 g	1921.4	<0.001	—	—	
	1500-2500 g			0.9	0.63, 1.26	0.535
	> 2500 g			0.52	0.35, 0.77	<0.001
Insurance		2.02	0.389			
Ethnicity	White (Ref)	65.84	<0.001	—	—	
	Asian			1.13	0.89, 1.45	0.319
	Black			0.68	0.53, 0.86	0.002
	Other			0.85	0.71, 1.00	0.057
Gender	Female (Ref)			—	—	
	Male	3.97	0.049	0.7	0.59, 0.84	<0.001
WBC		31.12	<0.001	0.99	0.97, 1.00	0.104
Basophils		0.03	0.980			
Eosinophils		5.94	<0.001			
Lymphocytes		16.96	<0.001	1	1.00, 1.00	0.093
Monocytes		6.11	<0.001	1	1.00, 1.00	<0.001
Neutrophils		1.61	0.107			
Basophils %		0.1	0.920			
Eosinophils %		5	<0.001			
Lymphocytes %		34.86	<0.001	1.02	1.01, 1.02	<0.001
Monocytes %		1.13	0.256			
Neutrophils %		34.22	<0.001			
Bands		14.94	<0.001			
Hematocrit		4.77	<0.001	1.24	1.03, 1.49	0.023
Hemoglobin		9.42	<0.001	0.55	0.32, 0.96	0.033
MCH		19.51	<0.001	1.34	1.04, 1.72	0.024
MCHC		14.78	<0.001			
MCV		26.01	<0.001	0.91	0.84, 0.99	0.024
Platelet		5.42	<0.001	1	1.00, 1.00	0.120
RBC		16.67	<0.001			
RDW		2.94	0.003			
TBIL		12.84	<0.001			

DBIL		10.1	<0.001			
IBIL		12.71	<0.001			
Antibiotic	No (Ref)			—	—	
	Yes	35.47	<0.001	1.13	0.96, 1.34	0.137
STAPH AUREUS COAG +	No (Ref)					
	Yes	77.58	<0.001			
Acetaminophen	No (Ref)			—	—	
	Yes	12.38	0.001	1.35	1.13, 1.63	0.001
Vancomycin	No (Ref)					
	Yes	180	<0.001			

Supplementary Table S2. Details of the logistic regression model.

Characteristic	OR	95% CI	p-value
GA			
≤ 28 weeks	—	—	
28-32 weeks	1.88	1.28, 2.78	0.001
> 36 weeks	0.06	0.03, 0.09	<0.001
32-36 weeks	0.39	0.25, 0.59	<0.001
Weight			
< 1500 g	—	—	
1500-2500 g	0.91	0.65, 1.27	0.590
> 2500 g	0.52	0.36, 0.76	<0.001
Ethnicity			
White (Ref)	—	—	
Asian	1.13	0.89, 1.45	0.314
Black	0.67	0.53, 0.85	<0.001
Other	0.84	0.70, 0.99	0.039
gender			
Female (Ref)	—	—	
Male	0.71	0.60, 0.84	<0.001
Monocytes	1.002	1.001, 1.003	0.002
Lymphocytes %	1.02	1.01, 1.02	<0.001
Acetaminophen			
No	—	—	
Yes	1.35	1.13, 1.62	0.001

Supplementary Table S3. Results for variance inflation factor (VIF).

	GVIF	Df	GVIF^(1/(2*Df))
GA	4.94208637	4	1.22106502
Weight	4.44143504	3	1.28209602
Ethnicity	1.08338545	3	1.01343796
Gender	1.40856614	1	1.18683029
Monocytes	1.02145447	1	1.01067031
Lymphocytes%	1.36984654	1	1.17040443
Acetaminophen	1.47573262	1	1.21479736

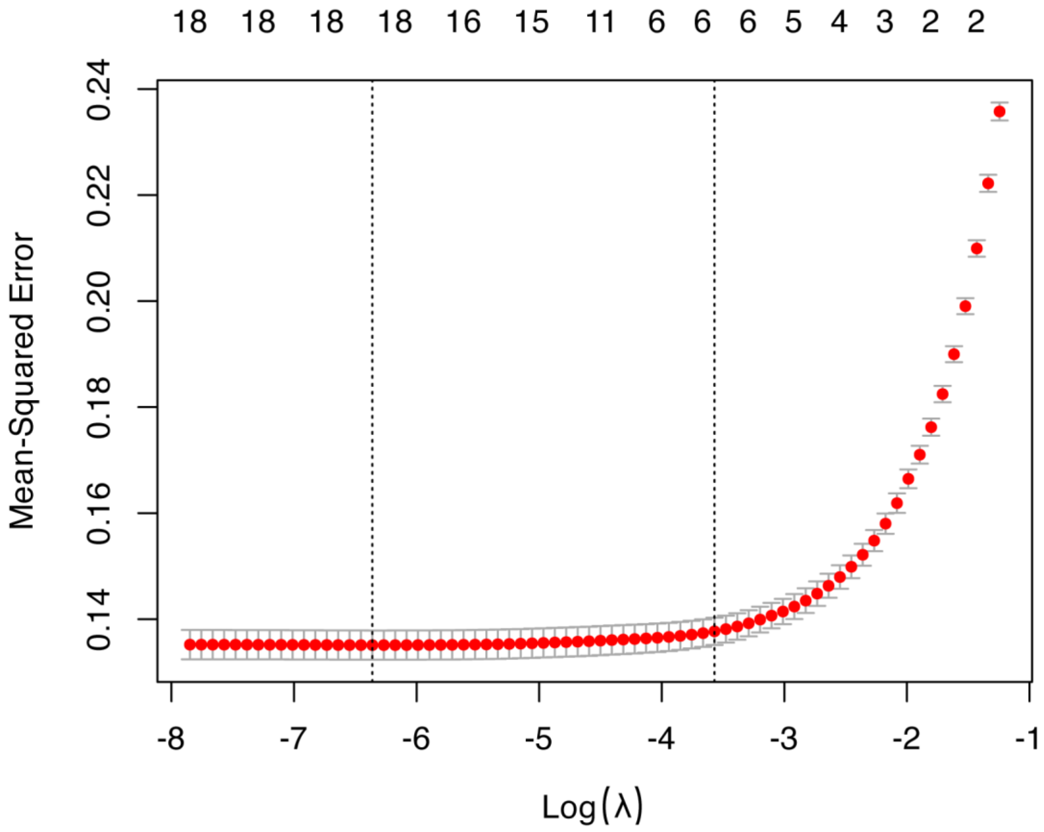

Supplementary Figure S1. LASSO regression cross-validation.
